# Breastfeeding practices among Syrian refugees in Turkey

**DOI:** 10.1186/s13006-022-00450-3

**Published:** 2022-02-14

**Authors:** Siddika Songul Yalçin, Meryem Erat Nergiz, Ömur Cinar Elci, Monica Zikusooka, Suzan Yalçin, Mustafa Bahadir Sucakli, Kanuni Keklik

**Affiliations:** 1 Unit of Social Pediatrics, Department of Pediatrics, Faculty of Medicine, Hacettepe University, Ankara, Turkey; 2 Department of Pediatrics, Yıldırım Beyazıt University, Yenimahalle Research Hospital, Ankara, Turkey; 3 Refugee Health Program WHO Country Office, Ankara, Turkey; 4 Department of Food Hygiene and Technology, Faculty of Veterinary Medicine, Selçuk University, Konya, Turkey; 5 Department of Migration Health, General Directorate of Public Health, Ministry of Health, Ankara, Turkey

**Keywords:** Syrian refugees, Teenage pregnancies, Breastfeeding, Anise, Short birth interval, Maternal nutrition, Pandemic

## Abstract

**Background:**

We evaluated (a) opinion of Syrian and Turkish healthcare workers (HCWs), and perceptions and attitudes of Syrian refugee mothers, pregnant women, fathers and grandmothers on age-appropriate breastfeeding, (b) the effect of cultural characteristics, migration and pandemics on Syrian’s infant nutrition, and (c) the suggestions of HCWs and Syrian family members to improve breastfeeding practices in the Syrian refugee society in a qualitative study.

**Methods:**

The qualitative study consisting of structured focus group discussions (FGDs) was held in four provinces in Turkey where Syrian refugees live intensely in September and October 2020. Seven different types of online FGDs were held with Turkish HCWs working in maternity hospitals, Syrian HCWs working in Refugee Health Centers (RHCs), Syrian pregnant women, mothers, fathers, and grandmothers. In total, we carried out 46 FGDs with 335 individuals. Thematic analysis of the transcripts in a deductive-inductive fashion was carried out with MAXQDA 11.

**Results:**

Most Syrian HCWs did not get any training on breastfeeding counseling. The short duration of breastfeeding in Syrian refugees was seen to be related to the cultural characteristics, and migration. Some cultural characteristics can be summarized as “believing that breastfeeding harms mother’s health”, “adolescent marriages”, “wanting to have as many children as possible”, “giving anise to infants and not breastfeeding at night”, “prelacteal feeding”, “believing that milk is not enough”, “over controlling mother–child interaction by grandmothers, which limits the interaction”, “short pregnancy intervals”, and “not using modern family planning techniques”. We found out that migration increased the tendency for adolescent pregnancies, deepened the poverty, and decreased family social support. We did not observe any change in breastfeeding practices during pandemics.

**Conclusions:**

Breastfeeding counseling programs should be designed in consideration of cultural characteristics of Syrian HCWs and family members. Continuing health education programs for family members with socially appropriate interventions to prevent adolescent marriages are important.

## Background

Breastfeeding is significant in achieving Sustainable Development Goals #2 and #3, which aim for ending hunger, promoting healthy nutrition and wellbeing [1–3]. The World Health Organization recommends that infants start breastfeeding within the first hour of life; they should be exclusively breastfed for six months, with timely introduction of adequate, safe, and properly fed complementary foods while continuing breastfeeding for up to two years of age or beyond [4]. Breastfeeding, as a fundamental right, must be promoted especially during high-risk conditions, where artificial feeding brings great risk to the child’s health and survival [5–8]. Disasters and refugee crises especially have an adverse impact on breastfeeding [9, 10].

Lactation is a natural process that starts without any external intervention. However, it has been reported that social and cultural determinants, personal habits have an impact on breastfeeding in human beings [11–20]. Breastfeeding studies among migrants and refugees report additional challenges [18]. Breastfeeding practices among Syrians refugees in Jordan were reported to be poor; early initiation of breastfeeding 32% and 29% exclusive breastfeeding (EBF) [21]. In addition, the observed EBF in Syrian refugees in Jordan (24.2%) was lower than the rate reported in Syria (42.6%) before the crisis [22]. However, early initiation of breastfeeding among Syrian mothers in Turkey was 61.4%; the EBF and continuing breastfeeding for 12 months were 28.1% and 55.0% respectively [23], and 24% of Syrian infants in Turkey received prelacteal feeding in Syrian Migrant Sample in Turkey [24].

Syrian refugees’ perceptions, attitudes, and socio-cultural factors about breastfeeding are not well known. None of the published literature was able to diagnose and explain the genuine reasons behind poor breastfeeding practices in Syrian refugees. Present studies included only mothers and mostly prevalence reports from one hospital or province. However, to effectively promote breastfeeding, the perceptions, attitudes and socio-cultural factors that influence breastfeeding practices in specific populations should be well understood. Among migrants and refugees in Turkey, Syrians have the largest fertility rate; about 10,000 Syrian babies are born monthly [25]. Therefore, we aimed: (a) to identify the opinion of healthcare personnel (HCWs), and perceptions, and attitudes of Syrian family members on age-appropriate breastfeeding, specifically early initiation of breastfeeding, and “continued breastfeeding for the first two postpartum years, (b) to identify cultural characteristics of Syrian refugees on infant nutrition practices and the effect of migration, (c) to evaluate the influence of pandemics on child care, (d) to discover the suggestions of HCWs and Syrian family members to improve breastfeeding practices in the Syrian refugees in a qualitative study.

## Methods

### Study type

This qualitative study, a joint project between WHO, The Ministry of Health (MoH), and the Hacettepe University, included structured, online focus group discussions (FGDs) [26]. The Ethical Review Committee approvals from Hacettepe University, the MoH, and WHO (ERC.0003291) obtained for the study.

### Study population

We used purposive sampling method and included four provinces, and two districts from each to ensure triangulation, reduce bias, and obtain a diverse information from participants.

We selected four provinces; Gaziantep, Hatay, İstanbul, and İzmir, which host about 31% of the Syrian refugees in Turkey [25]. With this selection, we aimed to include Syrian refugees with varying mobility and living conditions. Gaziantep and Hatay provinces are near the Syrian border and are entry points for refugees. Izmir is attractive for refugees due to agricultural workforce demand. Istanbul is a metropolitan city with an industrial infrastructure that hosts the highest number of Syrian refugees in Turkey.

**Fig. 1:**
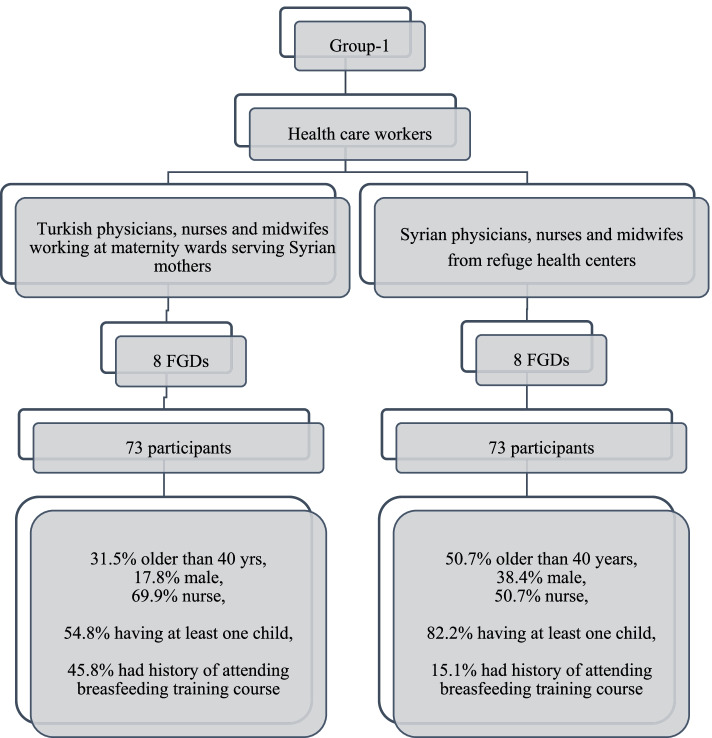
Characteristics of focus group discussions (FGDs) for healthcare workers (values are *n* or %)

We designed two distinct main groups with seven subgroups for FGDs;

Group 1 (Fig. 1): Two subgroups of HCWs in the selected provinces (a. Turkish physicians, nurses, and midwives from maternity hospitals serve Syrian mothers; b. Syrian physicians, nurses, and midwives from Refugee Health Center).

**Fig. 2:**
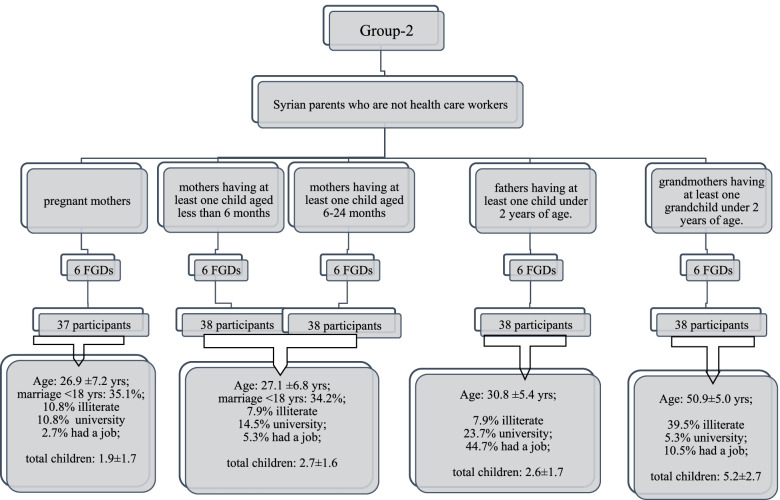
Characteristics of focus group discussions (FGDs) for family members (values are mean ± standard deviations, *n* or %)

Group 2 (Fig. 2): Five subgroups of Syrian family members (mothers having one infant younger than 6 months, mothers having one infant aged between 6–23 months, pregnant women, fathers having at least one child younger than 24 months, grandmothers having at least one grandchild younger than 24 months).

Exclusion criteria for family members: (a) Parents, whose children were cared for by someone else, (b) Family members whose relatives participated in previous FGDs, (c) Family members having at least one relative who were HCWs, (d) Family members working in the hospital or RHC as a secretary, interpreter, cleaner or driver.

### Topic guide questions

Topic guide questions for HCWs, mothers and fathers and grandmothers (Table 1) were prepared taking into account WHO indicators for infant feeding [27, 28] and other literature about breastfeeding [10, 12, 13, 29, 30].

**Table 1:** Thematic questions for FGDs

HCWs	Mothers; lactating or pregnant	Fathers, Grandmothers
• How do Syrian mothers feed their babies? • When do they first breastfeed their babies after birth? When is complementary feeding started? How many months are babies getting breast milk? What are the types of the preferred complementary food?	• Which infant’s feeding do you recommend for the healthy growth? • How long should a baby take breast milk for a healthy life? • When did you start breastfeeding your youngest baby after birth? How did you feed your baby in the first three days of his/her life?	• Which infant’s feeding do you recommend for the healthy growth? • How long should a baby take breast milk for a healthy life? • When did you start breastfeeding your youngest baby/grandbaby after birth? How did you feed your baby/grandbaby in the first three days of his/her life?
• What are the cultural characteristics for infant feeding in Syrians society?	• What are the cultural characteristics for infant feeding in Syrians society? • Can your child’s gender, birth order or illness affect his/her breastfeeding status? • What is the effect of immigration from Syria on breastfeeding?	• What are the cultural characteristics for infant feeding in Syrians society? • Can your child’s gender, birth order or illness affect his/her breastfeeding status? •What is the effect of immigration from Syria on breastfeeding?
• What are social and family support for breastfeeding?	• Have you received support from any person (your healthcare provider / relative) about breastfeeding? What did your relatives and your husband advise you about infant feeding or breastfeeding? • What was the impact of fathers, grandmothers and health personnel on breastfeeding?	• What are social and family support for breastfeeding?
• What is the value of breastfeeding in the Syrian culture?	• What is the value of breastfeeding in the Syrian culture? • How can breastfeeding affect infant and maternal health?	• What is the value of breastfeeding in the Syrian culture? • How can breastfeeding affect infant and maternal health?
• What are the barriers to the continuity of breastfeeding?	• What are the difficulties you encounter in the continuity of breastfeeding?	• What are the barriers to the continuity of breastfeeding?
• Has the corona pandemic affected childcare, and feeding type of Syrian babies?	• Has the corona pandemic affected your childcare, and the way you feed your baby?	• Has the corona pandemic affected health and feeding of your baby?
• Do you have a breastfeeding counseling certificate? If yes, are there any factors that challenge you when providing breastfeeding counseling / training to Syrian refugees unlike the local population? What are they?		
• Scenario Technique: “International associations including the World Health Organization, UNICEF recommended that babies should be breastfed for at least two years; How can we achieve this with Syrian babies to breastfeed them for at least 2 years?”	• Scenario Technique: “International associations including the World Health Organization, UNICEF recommended that babies should be breastfed for at least two years; How can we achieve this with Syrian babies to breastfeed them for at least 2 years?”	• Scenario Technique: “International associations including the World Health Organization, UNICEF recommended that babies should be breastfed for at least two years; How can we achieve this with Syrian babies to breastfeed them for at least 2 years?”

Topic guide questions were written in English and translated into Arabic; they were retranslated back into English to ensure reliability. Then, they were edited by scientific reviewers and WHO Ethical Review board. The pilot implementations of FGDs with mothers and Syrian HCWs were performed face-to-face in Ankara in September, 2019. Thus, the topic guide questions were tested and improved.

### Study procedures

The MoH provided written communication about the study and the contact information for the research team to selected hospitals, and Refugee Health Centers (RHCs) from the selected provinces and requested the invitation of targeted participants to take part in the study. A volunteer HCW from each RHC announced the study, selected the family members to participate among the ones who agreed and met inclusion criteria. FGDs were conducted in Turkish for local HCWs and in Arabic for Syrian HCWs and refugees; female interpreters for female refugees and male interpreters for male refugees interpreted FGDs.

All FGDs were conducted in September and October 2020. Zoom link was sent to HCWs before the meeting and they attended the FGDs where they wanted, whereas voluntary family members were called to the RHCs at the appointed time to provide a safe, private environment that families could access easily. Participants signed informed consent forms before the study. During FGDs, all personal identifiers of participants were masked by nicknames. All data including a short socio-demographic questionnaire and FGDs collected using these nicknames only. A nurse in selected RHCs ensured that the zoom link was established, the nickname of the participant written on screen and the family participant was connected with RHC’s computer. One participant per family attended the FGDs. One appointed HCW in each hospital and RHC collected the file including the signed informed consent and filled questionnaire and sent images of the file using WhatsApp.

Due to the COVID-19 pandemic, we used the Virtual FGDs with audiovisual software. We selected the ZOOM platform, because of its wide diffusion, intuitive interface, and interactive functions. To allow everyone sufficient time to express themselves, we limited the number of participants, into 6–10 participants for each 60–90 min FGDs. Interviews were audio-recorded with the nicknames.

Each FGD session was performed by two researchers, supported by bilingual translators. SSY, an experienced qualitative researcher, moderated the meetings. The second researcher (MEN) was trained for collection techniques and all FGDs were performed with the same structured open-ended questionnaire. Both researchers, took notes regarding study participants’ verbal and non-verbal cues during sessions of FGDs and achieved intersubjective agreement for observer triangulation.

Prior to FGDs, bilingual translators (*n* = 4) were trained on the study subject including standardized research questions. Controversial expressions in the translation were also checked by the other two translators through audio-recordings’ data and a consensus were achieved.

A pre-packed bottle of water before the meeting and “sanitation and hygiene kit” at the end of the meeting were provided to each FGDs’ participant.

### Trustworthiness

Triangulation was used to counter all threats to trustworthiness; reactivity, researcher bias, and respondent bias. Two observational data sources (written notes for gestures [facial expressions] and non-verbal expressions such as laughing, coughing, groaning) and transcript of audit data were included to ensure data triangulation. Two coders were present for analytic triangulation and the categories and themes that emerged were confirmed by inter coder consensus.

### Data analysis

First, one researcher (MEN) anonymously transcribed audio-recordings verbatim. The text transferred to software and then two researchers (SY, SSY) read the text while listening to audio-recordings for any mistake and to proof-read. Three researchers (MEN, SY, SSY) at team meetings discussed differences in data and modified the transcription if necessary.

Thematic analysis of the transcripts was carried out. Categories, codes were developed in a deductive-inductive fashion. SSY constructed a codebook, and linked in categories (conceptual narrative labels) prior to data collection and modified it while in the field. Two coders (MEN and SSY) independently analyzed the text in the light of research questions, read the text over and over, highlighted central terms and concepts, marked and wrote memos. Then, two coders either assigned the code to every segment of text or identified recurrent themes and defined appropriate codes and associated the text passages with them. The third researcher (SY) read the text line-by-line, checked the codes for similarities and differences and noted every discrepancy between MEN and SSY, and used team meetings to discuss these discrepancies and to make decisions about which way the data should be coded. ‘Consensual coding’ was done to ensure that all the coders (MEN, SY and SSY) agreed in their understanding of how to apply the category system. Some codes were grouped together according to their similarity, while other codes started out too broad and were sub-divided. When there was no need for additional categories, the category system was fixed. As the data was compiled, data saturation was checked and cross-checked with the previous FGDs and it was seen that the data saturation was achieved at the end of the study. Some statements were selected to give their core with thematic summaries. Coding and analysis were performed using MAXQDA 11 (VERBI Software, Berlin, Germany).

## Results

There were a total of 335 participants (262 Syrians, 73 Turkish) to FGDs. Overall, 15.1% of Syrian HCWs and 45.8% of Turkish HCWs indicated that they received a WHO-Breastfeeding counseling training (Fig. 1).

One-third of mothers were married before 18 years of age. Overall, 8.5% of parents and 39.5% of grandparents were illiterate. Demographics of mothers, pregnant women, fathers, and grandmothers participating to FGDs were given in Fig. 2. In FGDs including 76 mothers, 14.5% (*n* = 11) had two children under two years old, three mothers were pregnant and current breastfeeding rate was 77.6% the last child. From 37 pregnant women, nearly half (*n* = 15) had one child younger than 24 months of age and only three of them were breastfeeding their last-born child. In the fathers’ FGDs, nine fathers had two children younger than 24 months and the current breastfeeding rate was 65.8% for the last child. In the grandmothers FGDs, the current breastfeeding rate was 81.6% for the last grandchild.

### Theme 1. Attitudes of Syrian refugee mothers to feeding their infants

Most HCWs stated that Syrian mothers were generally breastfeeding their infants. Half of the RHCs staff reported that Syrian mothers breastfeed due to financial difficulties but started complementary food as early as 2–3 months of age.

*“Usually mothers prefer breastfeeding because they are under the protection of Turkey, their finances are tight, and breast milk is less costly and more natural. (Gaziantep, RHC physician, male, 50–59 year old [y])”*

Nearly, all mothers in FGDs reported that they gave breast milk to their infants for some period of time. However, they claimed that sometimes when breast milk was not enough or the baby won’t latch, they supplemented with infant formula or cow’s milk before 4 months of age.

*“I want to breastfeed for 2 years, I plan like that but as he is not full with my breast milk currently I am both breastfeeding and bottle feeding, I want but I can’t. (Hatay, mother’s FGD, 20 y, 3 children, last child: 2 month old [mo], Currently breastfeeding [BF])”*

Most pregnant women planned to give breast milk after birth. Those with previous poor breastfeeding experiences or with twin pregnancies had anxiety about breastfeeding.

#### Early initiation of breastfeeding and prelacteal feeding

Nurses and midwives at maternity hospital stated that they give babies to mothers as soon as possible and encourage breastfeeding in the maternity hospital. The majority of HCWs reported that Syrian mothers gave colostrum to their infants, whereas, some mothers tried to give the baby something other than breast milk in the first three days after birth. Some HCWs reported that mothers with low pain thresholds and a tendency to sleep more preferred to feed their infants with sugary water and infant formula replacing breastfeeding.

Mothers and grandmothers stated that all mothers give colostrum. However, only one-third said they exclusively breastfed for the first three days. Some family members believe the milk won’t be enough the first three days; so, the baby should be supported. In addition to breastfeeding, the first three days after birth Syrian mothers also give supplementary food, such as sugary water, packaged fruit juice, infant formula, anise, dates, honey, cumin, and Zamzam (religiously blessed plain water).

*“I breastfed 1 hour after birth. Then, for the first 3 days, I gave cumin mint chamomile tea with sugar water. (Gaziantep, mother’s FGD, 37 y, 5 children, last child: 23 mo, Not BF)”*

*Since the baby could not learn how to suck on the first day, I started breastfeeding on the second day. I also gave sugary water in the first three days. (Gaziantep, mother’s FGD, 20 y, 1 child, 5 mo, Currently BF)”*

Those pregnant women who had given prelacteal feeding in their previous deliveries said that they will do it again because they will face problems like low milk supply.

*“Since the mother is tired and in pain after birth, we give sugary water to the baby. We boil anise, or we can boil something with dried herbs like chamomile or mint. Anise also allows baby to sleep calmly and peacefully. (Hatay, pregnant women FGD, 26 y, 4 children, last child: 16 mo, Not BF).”*

#### Complementary feeding

Most Syrian participants stated that Syrian mothers tend to start complementary foods as early as 40-days after birth with foods such as rice water, diluted cow’s milk, pudding, yogurt, tea, or boiled fruits. Especially where mothers work in agriculture, grandmothers care for babies and start complementary foods early.

*“Right after 40 days of birth, I gave yoghurt in the form of ayran and started to make him taste the meals little by little. (Gaziantep, Mother FGD, 37 y, 5 children, last child: 23 mo, Not BF)”*

In one-fifth of babies, complementary nutrition could be delayed until the age of one. Those who are in a good financial situation want their babies to be chubby and they use infant formula. Although most Syrian mothers start complementary feeding early, bottle feeding and late weaning are also prevalent. Boiled rice, boiled fruits, cow’s milk, black tea was among the most common complementary foods. In addition, babies were not allowed to feed themselves until after 18–24 months.

*“Now my baby is 13 months old. I recommend breast milk, I also breastfeed. I have started complementary foods for more than 2 months. (İstanbul, mother’s FGD, 24 y, 3 children, last child: 13 mo, Currently BF)”*

#### Breastfeeding duration

HCWs from RHC indicated that they observed the breastfeeding duration among Syrian mothers is shorter than 24 months, even 12 months, mostly, due to the short interpregnancy interval. Syrian mothers usually stop breastfeeding at the age of 6–18 months. Only a few breastfeed until the age of two.

One-third of Syrian mothers believed that babies should be breastfed until the age of two, and one-third suggested breastfeeding could be stopped at 18 months. Even, some mothers thought that “breastfeeding longer than 12–15 months is not correct”, “in case of new pregnancy breastfeeding should be terminated”.

*“I didn’t breastfeed my son. I will not breastfeed my daughter up to the age of one and a half, that would be too long. I think it will be more beneficial if I breastfeed until the age of one and then switch to home meals. (İzmir, mother FGD, 19 y, 2 children, 10 day old, Currently BF).”*

*“Doctor, I will not tell you what it takes for a baby to be breastfed until the age of two, because in my opinion, a child should not be breastfed until the age of two. Because if a child gets used to breastfeeding, he or she will not be inclined to additional foods. This will ensure that he does not sleep comfortably or straight, so it will be a problem. (İstanbul, pregnant women FGD, 32 y, 2 children, last child: 6y)”*

Some fathers believed that babies should be breastfed until the age of two years, but their wives stop breastfeeding before the 12th month. In addition, one father established an interaction between breastfeeding duration and teeth development.

*“Of course, it is normal for us not to continue for up to two years, because at the age of one and a half, all teeth are grown, the child bites the mother’s breast. (İzmir, Father FGD, 36 y, 5 children, last child: 2 mo, Not BF).*

Half of the grandmothers stated babies should be breastfed until the age of two, and the other half, until the age of 1.5 years.

### Theme 2: The cultural characteristics for infant feeding in Syrian society

#### Traditional practices and breastfeeding

Syrian HCWs and family members indicated that most parents in Syria traditionally believe sugary water is nutritious and it cleanses the intestines and prevents jaundice. Anise is used for nutrition, pain relief and to calm infants to sleep. Anise is also given to reduce mother’ pain and increase milk production just after birth. More than half mothers and grandmothers reported that night sleep is very important for both mother and baby and this situation is supported by preparing special herbal teas; mostly anise, some cumin, papaya tea and even a mixture of them. It was seen that breastfeeding at night was not desired even in the neonatal period.

*“We give breast milk to the baby after birth and give very little sugar water to prevent jaundice.* *(Hatay, grandmother FGD, 53 y, 6 grandchildren,* *last child:* *8 mo, Currently BF)*”

“*For the first 3 days, it is necessary to give sugary water with breast milk so that the intestines are cleaned.* *(Hatay, grandmother FGD, 50 y, 4* *grandchildren,* *last child:* ”

*“We give sugary water because breast milk does not come immediately after birth. When the breast milk comes, we immediately give the child to the mother's breast and breastfeed, but we do not breastfeed too much so that jaundice does not occur. (İzmir, grandmother FGD, 47 y, 6 grandchildren, last child: 1 mo, Currently BF)”*

Some HCWs at hospital stated that their babies were born with religious fasting and they broke the fasting by giving babies a piece of dates.

*“I saw several times that they rub dates in babies mouth before breast milk. When I asked why, they even said something like babies should break the fast with this. (Hatay hospital-nurse, female, 40-49 y).”*

Most Syrian HCWs and family members participating FGDs reported that infant’s gender or birth order did not influence his/her breastfeeding status. However, few mothers said that female infants are breastfed less because they want them to be thin and more delicate. Some HCWs and mothers stated that the first-born children were breastfed for a shorter period of time because they wanted the second to come immediately and to have more children.

#### Postpartum strict social support from grandparents, relatives

Some nurses and midwives at maternity hospitals reported that social support among Syrians, especially for adolescence pregnancies can sometimes be excessive; other family members provide care for the baby so that the mother can rest, which might reduce contact with the mother and prevent mothers’ attachment to the baby.

*“Babies are always in the arms of their relatives. They do not attach their babies much, they do not smell. Therefore, they cannot adapt to their babies. (Gaziantep hospital-nurse, female, 30-39 y).”*

#### Bottle-feeding and wealth

Some mothers believed that when the child cries, the mother should quit all her work and breastfeed. Therefore, this prevents mothers from doing their work and steals time from other children and husband. Yet, they suggested everyone who can hold a bottle could feed the baby. They also complained that breastfeeding disrupts the night sleep. Most mothers feel shy to breastfeed outside and preferred to use a bottle when they go out.

*“Bottle feeding provides comfort to the mother. Especially when she goes out, she can leave the ready-made milk in the bottle and go. If a mother is a clean person, by God’s willing, nothing will happen; but, if the mother is not clean, anything can happen to that baby. (Hatay, grandmother FGD, 53 y, 2 grandchildren, 15 day old, Currently BF)”*

#### Maternal undernutrition, adolescence pregnancies, short birth intervals

Most Syrian HCWs and Syrian parents in FGDs reported that the biggest reason to limit breastfeeding was “malnutrition and fatigue of the mother and a new pregnancy”. The biggest obstacle to breastfeeding was short birth intervals and adolescent pregnancies. Adolescent mothers lack basic nursing skills, they cannot adapt to motherhood and accept the baby. Most Syrian family members believed that breastfeeding should be discontinued in case of new pregnancy.

*“The Quran says up to two years, of course this is the right of the child, but the mother also has a right. For example, if she gets weak after breastfeeding for a year and two months, or if she has a pregnancy, then she should stop breastfeeding. (İstanbul, Father FGD, 26 y, 2 children, last child: 18 mo, Currently BF)”*

#### Opinions for short breastfeeding duration and proposed reasons

Most parents used negative statements when discussing breastfeeding until 24 months. They think that it is unnecessary, it will weaken the mother. Overall, parents believed that breastfeeding after 18–24 months will cause the child to become dependent on the mother, reduce appetite and prevent adequate nutrition.

*“In the past, in our mothers' time, there were no such infant formulas. I think it's very wrong to breastfeed for 2 years nowadays. I think so, at least 6 months to 1 year will be enough. I saw it in my nephews. They become weaker as they rely on their mothers for food. Mothers cannot do anything to them either. (İzmir, pregnant women FGD, 40 y, first pregnancy)”*

#### Breastfeeding in Ramadan

Although it is not mandatory for women who are breastfeeding to fast in Islam, almost all mothers in FGDs claimed they fast in Ramadan both during pregnancy and breastfeeding periods. They stated that they could start fasting after the baby was 40-days old, but they also admitted that during Ramadan the mother becomes weak and even the milk production stops. But regardless, they continue fasting.

*“I had fasted in Ramadan when I was pregnant, I had no problem. But after fasting a while, breast milk became insufficient and then it stopped and when she was 2 months old; I had to return to bottle. (Hatay, Mother FGD, 37 y, 7 children, 1 mo, Currently BF)”*

#### Maternal self-care and husband value

According to both Syrian and Turkish HCWs, Syrian mothers have aesthetic concerns; they are afraid that their husband will bring another wife and consider the needs of their spouse before those of the infant. Some nurses and midwives from maternity hospital stated that mothers who have just given birth pay great attention to their self-care and try to look beautiful for their husband.

“*Syrian mother wears makeup in front of the mirror, because she will meet her husband. She wears special clothes*. *(Istanbul, hospital-nurse, female 30–39 y)*”

*“I observe a situation that mothers prefer their husbands a little more than babies. When I visit them at the ward, they ask after their husbands after the delivery. They refuse to breastfeed the newborn and tell me that they need their husbands not the baby. (Istanbul, hospital-nurse, female 30-39 y)”.*

#### Breastfeeding and family income

The HCWs reported that mothers from higher-income families tend to use infant formula and those with a lower income are forced to breastfeed for financial reasons.

“*High-income mothers tend to give infant formula. Other mothers have to breastfeed because they cannot get formula*. *(Izmir, hospital-nurse, female 40–49 y)*”.

While one-third of the pregnant women did not think that breastfeeding had any effect on family dynamics, half of the pregnant women believed that breastfeeding supports the family’s economy.

#### The impact of fathers, grandmothers, and health personnel on breastfeeding

Delivery room nurses and midwives stated that they tried to give breastfeeding counseling with an interpreter and body language. However, when they turned their backs, the grandmother had taken the child from the mother. They observed that the frequency of milk insufficiency is less. In addition, they stated that Syrian mothers did not report breastfeeding difficulties and did not ask for help.

While most of the mothers reported the support of grandmothers on breastfeeding, some received support from husbands, relatives, and their older female children. One-fifth did not have any support. These mothers complained that they immigrated with their husbands, their families remained in Syria and they could not find anyone to consult with. Only a few reported that they consulted the HCWs when they have breast-nipple problems. Five mothers stated that they were also trying to solve their problems through resources on the internet.

#### Decision to breastfeed

All Syrian family members in FGDs stated that mothers decided the duration of breastfeeding. Since “breastfeeding is a big burden” on the mother, and it might affect her health, this right belongs to the mother. They stated that “like all household chores such as cooking, cleaning, the duration of breastfeeding should be determined by the mother as she is the ‘manager’ of the house”. Some mothers stated that they consulted their husbands, mother-in-law and their mothers. Despite the fact that a few mothers claimed that their husbands and mothers pursued them on continuing breastfeeding, all Syrian participants claimed that the final decision is with the mothers.

*“The father shouldn't have much to do, this topic is a matter of the mother’s decision, I think the father shouldn't have anything to do, as the mother knows how long her body can endure. (İstanbul, pregnant women FGD, 23 y, 1 child, 16 mo, Not BF)”*

*“In my first child, my husband asked me to breastfeed until the age of two but when the child was a year and a half old, my body grew lean, I became weak, we also entered the month of Ramadan so I had to wean but I don't know what will happen with my second child. (Hatay, pregnant women FGD, 18 y, 1 child, 19 mo, Not BF)”*

### Theme 3. The perception for the value of breastfeeding in the Syrian culture

#### Infant health benefits

Almost all Syrian parents in FGDs believed breast milk is important for a baby’s nutrition. All family members know that breastfeeding has positive effects on a baby’s health; they got sick less, they grow and develop better.

*“In fact, we compare breastfeeding for up to two years to a growing tree. In other words, a child who does not suck for two years is like a tree that does not give fruit, in fact, when we wean breastfeeding, there is a lot of nutrient deficiency. I remember, for example, I was breastfed until the age of four or five, I even remember that period, I do not know that I have been to the hospital so far (laughing). (İzmir, Father FGD, 36 y, 5 children, 2 mo, Not BF)”*

*“Breastfeeding is of course a good thing, it is rewarding. It will have a beneficial effect on the baby. If the mother is well-fed, the baby is also fed from the milk. (Hatay, Mother FGD, 18 y, 1 child, 18 mo, Currently BF)”*

Some mothers believe that breast milk may not work the same way for all babies.

*“Breast milk is good for the baby and they should get until the age of 1.5. But mother’s psychological status is important; if they don’t feel good, babies might be affected, they might get sick, they might get diarrhea. (İzmir, pregnant women FGD, 21 y, 1 child, 4y).”*

#### Attachment

All family members stated that breastfeeding has a positive effect on the attachment between mother and baby.

*“At first, I didn't like breastfeeding, I had a hard time. But when I saw that we established a connection with the baby, I started liking it very much. (Gaziantep, Mother FGD, 20y, 1 child, 5 mo, Not BF)”*

#### Maternal health concerns

Some Syrian HCWs from RHCs and most family members believed that breastfeeding does not have a positive effect on maternal health; they believed it weakens and tires the mother; it causes maternal tooth decay and hair loss. A few mothers complained that breastfeeding caused the mother's breasts to get bigger, asymmetric, and sag.

*“Breastfeeding a child is a little difficult for me, my teeth broke and I lost a lot of weight because of this. But on the other hand, it helped my child a lot, it helped my child's development, but it was very harmful to me. (Istanbul, Mother FGD, 34 y, 2 children, last child: 19 mo, Not BF)”*

*“ . . . you see how many times the baby sucks during the day. That's why the mother will be tired as the baby pulls everything from her body, so she needs to pay attention to her nutrition. If she pays attention, her problems will decrease; otherwise breastfeeding will of course tire a mother too much. (Hatay, Mother FGD, 27 y, 2 children, last child: 23 mo, Not BF)”*

*“I am now six months pregnant and I'm still breastfeeding my nine-month old baby. Of course, my baby is in a very good condition as I give him a healthy breast milk. However, foregone conclusion, I get tired, I am affected and weak. Actually, I am thinking of weaning, but I cannot do anything because the baby has gotten so used to it. (Hatay pregnant women FGD, 24 y, 2 children, 9 mo, Currently BF)”*

### Theme 4. Being a refugee

Turkish HCWs believed that the Syrian refugees instinctively reproduced more because they fled the war and suffered many losses. They said that the depression, economic difficulties and communication problems due to immigration affect breastfeeding negatively. They reported that they could not communicate due to language problems and that the interpreters were insufficient. They said that mothers do not want to talk about special issues such as contraception through a male interpreter, so there is a need for female interpreters.

“*I think Syrians tend to reproduce because of the war in their country, because of human losses. (İstanbul hospital-nurse, female, 20–29 y).*”

*“ . . . but I think they came to a foreign country by immigration, so they may be suffering from depression. (İstanbul hospital-*physician*, female, 50-59 y).”*

*“I think the most serious problem is the lack of interpreters. The majority of interpreters in the hospital are male, so they are rightly hesitant to talk about these issues (contraception). (Gaziantep hospital-*physician*, male, 50-59 y)”*

Syrian HCWs said that some refugee mothers, on top of household responsibilities, had to work due to financial difficulties. Especially the mothers working in agriculture started to work as soon as possible after giving birth and spent almost half of the day at work. Therefore, they said that they could not breastfeed for a long time. They said that children could not go to school due to economic difficulties and security reasons, which resulted in bringing the age of marriage earlier than already young age of marriage in Syria after migration. Consequently, increasing adolescent pregnancies and lower education level make breastfeeding difficult. In addition, they reported that the psychological distress and loss of social support as a result of migration adversely affected breastfeeding.

*“In Syria, women were not working outside the home, but now they are working nearly 10 hours a day and they cannot pay attention to breastfeeding. (Istanbul RHC-*physician*, female, 30-39 y)”*

*“Previously, mothers were breastfeeding but because of the economy, early marriages, living alone, having no grandmothers, not getting support, being too young, bad nutrition of the mother, no breast milk comes after delivery, how can a baby to be breastfed in this case. (Hatay RHC-nurse, female, 20-29 y)”*

On the other hand, mothers mostly complained about the loss of social support, in addition to economic difficulties and adjustment problems.

*“Regarding the nurturing of the baby, the mother is the same here as in Syria. Mom doesn't change. We are only experiencing social difficulties; we are having a lot of trouble caring our children here. One's own homeland would be different, we are experiencing a social problem here. We experience difficulties even in our behavior towards our children due to the environment. (İstanbul, pregnant women FGD, 26 y, 2 children, last child: 6 y)”*

*“When I was in Syria, I would immediately ask my mother or mother-in-law if the child would cry a little. I used to go to them before the doctor for my child, but they are not here. (İstanbul, pregnant women FGD, 32 y, 2 children, last child: 5 y)”*

### Theme 5. Possible ways to support breastfeeding of Syrian babies for at least 2 years

To support breastfeeding, the first suggested solution by Syrian HCWs was “training” HCWs and pregnant mothers, admitting spouses and grandmothers in pregnancy training schools and providing Arabic training with female interpreters. Most Syrian HCWs from RHCs did not have breastfeeding counseling training and they would like to receive training.

Another proposed solution from both Syrian and Turkish HCWs was providing family planning. Improving maternal health services, eliminating communication problems using reward-punishment methods were other recommendations.

*“Although the contraception is rejected as a sin, and the parents do not know much about it, they still believe that they can use contraception if it is explained. (Istanbul, hospital-nurse, female 30-39 y)”*

*“I would suggest this, I would stop unconditional aid. For example, the “Kızılay” (Red Crescent) distributes aid cards. I wouldn't give those cards unless mothers provide a minimum of two years of breastfeeding. (Hatay RHC-physician, female, 30-39 y)”*

Among the suggested solutions from Syrian parents’ FGDs, the most emphasized was “improving the health of the mother”, that the mother should eat well, pay attention to what she eats and take her vitamins.

*“If the mother's nutrition is not good, the milk she gives will not only harm the baby, but also harm herself because the mother is not well-fed. I had to stop breastfeeding because I had nutritional shortage; when I stopped breastfeeding some of them were in six months or in nine months or 15 months. In such cases, we were buying milk from the pharmacy. (İzmir, pregnant women FGD, 39 y, 6 children, last child:11 y)”*

When asked for suggestions for a short gestational interval, “family planning” was the first solution. While most of them used the traditional contraception methods such as withdrawal, only a few stated that they know about and use modern methods.

### Theme 6. Influence of pandemic on child nutrition and healthcare

HCW in hospitals stated that the number of visits by Syrian mothers to maternity hospitals, which is less than recommended in pregnancy follow-up protocols in Turkey and limited with emergencies and delivery, did not change due to COVID-19. A doctor in hospital indicated that nothing has changed since the pandemic, the aware ones continued to come. Low number of antenatal follow-up visits resulted in fewer training sessions, including breastfeeding provided for mothers. Almost all HCWs in hospital reported that Syrian mothers wanted to be discharged immediately due to the fear of COVID-19.

Syrian HCWs at RHCs said that at the beginning of the pandemic, families hesitated to visit the center, they postponed their children's vaccinations and routine follow-ups, but two or three months later they were back again to catch up with the missing vaccinations and follow-ups.

*“In the first 3 months, the follow-ups decreased a lot. Usually, there was a 1-2 month delay in the vaccinations of children. Initially mothers were afraid, but then they started to come. (İzmir RHC--doctor, male, 20-29 y )*

Most of the fathers had financial difficulty, there was a shortage of food in their houses, and they faced problems buying complementary foods.

Most mothers stated that they were afraid to leave the house during the pandemic, but they took necessary preventive measures and continued going to hospitals for childbirth and vaccination. They claimed to have difficulty in getting an appointment in hospitals. Therefore, they prefer to visit RHCs. They reported that when the RHCs were closed, they visited private Syrian doctors and purchased medicines from other Syrians when needed. A few people said that because of financial difficulties and because they stay at home for longer periods, mothers tend to breastfeed more. Some Syrian family members reported that during the pandemic breastfeeding or infant feeding was not affected. Only one mother stated that her breast milk production was reduced due to poor access to food.

“*The infants' feeding was not affected. Their follow-up to hospitals decreased but they did not delay their visits to RHCs*. *(Hatay, RCH-nurse, female 30–39 y)*”.

*“We couldn't take the babies for vaccinations at first, but after the disease started to decrease, we did. When we needed medicine, we bought it from Syrians (not legal). (İzmir, Mother FGD, 19 y, 2 children, 13 day, Currently BF)*

## Discussion

**Fig. 3:**
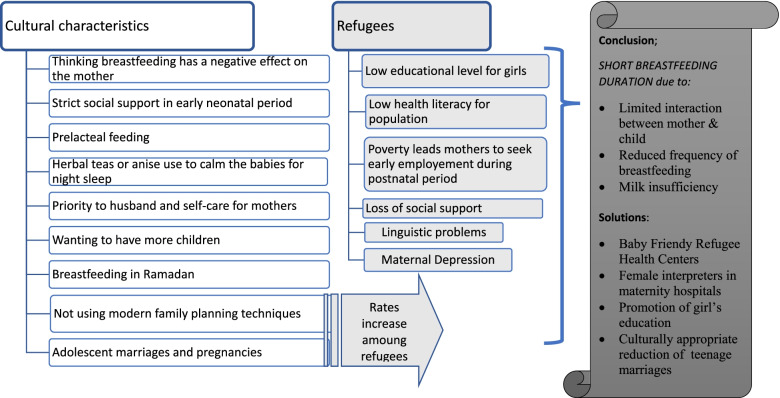
Determinants of short breastfeeding duration and how to support breastfeeding in Syrian refugees in Turkey

According to the information we obtained from HCWs and families, the breastfeeding problems in Syrian immigrants are as follows (Fig. 3): a) mixed feeding from birth, starting complementary feeding too early or later than the ideal time, and a short total breastfeeding duration. The cultural characteristics associated with all these breastfeeding problems are a) Thinking breastfeeding has a negative effect on the mother b) Over-controlling mother–child relation by grandmothers in early neonatal period c) Prelacteal feeding d) Herbal teas or anise use to calm the babies for night sleep e) Breastfeeding in Ramadan f) Priority to attending husband and self-care for mothers g) Wanting to have more children h) Not using modern family planning techniques and i) Adolescent marriages and pregnancies. In addition to those it has been reported that the COVID-19 pandemic has no effect on breastfeeding or infant feeding. As a solution to breastfeeding problems in Syrian mothers, we can suggest a) Baby Friendly RHCs b) Culturally appropriate healthcare in maternity hospitals c) Promotion of girls’ education d) Culturally appropriate reduction of teenage marriages.

This study observed that EBF up to 6 months and continued breastfeeding up to 24 months were rarely practiced among Syrian refugees. Infants are exposed to food without essential nutrients for growth and development. Similarly, Demographic Health Survey reported 13.7 months median duration for breastfeeding and 3.1 months for EBF among Syrian refugees in Turkey [24]. Ozkaya et al. also reported a shortened breastfeeding period in refugee women from Syria [31].

Cultural characteristics, migration, and low health literacy were found as main risk factors for short breastfeeding duration (Fig. 3).

The total fertility rate among Syrians in Turkey is 5.3 births per woman [24]. Similar to our study, nearly 38% of Syrian women have births intervals less than 24 months in TDHS [24]. As reported in previous studies [19, 32, 33], short birth interval, which is linked to “lack of modern family planning techniques”, and “wanting to have many children”, is associated with poor breastfeeding practices in our study. For Syrian families, children mean power and children are accepted as “God-given”.

Despite the known benefits of breastfeeding to mother and baby [3, 34], most Syrians perceive that breastfeeding has a negative impact on the mother’s health, and breastfeeding decisions are based on traditional knowledge, myths, and misinformation, similarly with the previous studies [35–37]. Regardless of the education and parity, they follow traditional beliefs and practices as reported earlier [23, 33, 38, 39]. Prioritizing a mother’s self-care, the admiration of her husband, and strong mother-in-law control might adversely affect breastfeeding decisions and limit maternal-child bonding [35].

Syrian mothers give anise to babies as a prelacteal feed and continue to give anise to control crying, which is perceived as tiring, and to promote night sleep. Prelacteal feeding and reduced nocturnal breastfeeding might hamper the cycle and the quantity of prolactin secretion, which reduces milk production and the suppressive effect of lactation on ovulation. Feeding herbal tea to soothe infants is a prevalent behavior in Arab communities [40, 41].

We observed that mothers prioritize fasting during Ramadan, and this might lead to dehydration of mothers and reduction of breast milk [42].

Adolescent marriages are prevalent with low maternal education, low health literacy, and poverty among refugees. Among Syrian women in Turkey, the prevalence of adolescent pregnancy is 40% [24]. Although adolescent marriage was common in the pre-war Syrian community, after the war, to provide so-called safety and security, teenage girls were allowed to get married earlier than before [43–45]. However, adolescent mothers could not provide necessary care and attention to their babies [46]. Previous reports also showed that Syrian refugees are at risk for inadequate antenatal care, adolescent pregnancy, and adverse pregnancy outcomes [47, 48]. In addition, nutritional inadequacy was identified in higher proportions of lactating and pregnant Syrian refugee mothers in Turkey and Lebanon [48, 49]. Promoting the education of girls will greatly contribute to the healthy nutrition of Syrian children by both increasing health literacy and preventing adolescent marriages.

It was observed that the effect of social support on breastfeeding among Syrian immigrants changed according to the age of the baby. However, previous studies reported social support in mother migrated from Syria affected duration of breastfeeding and age-appropriate feeding attitudes in Turkey [31, 50]. In addition, similar to our study, maternal depression were reported to be high in Syrian refugee mothers in Lebanon [51] and Turkey [52].

Culturally appropriate health education supported by social norms is necessary to support breastfeeding among Syrian refugees in Turkey (Fig. 3). Mothers are the decision-makers on breastfeeding; changing their negative perceptions about maternal health is important. Initiatives that promote breastfeeding and emphasize the value of breast milk should be organized. Behavioral change strategies are needed to encourage mothers to adopt the right breastfeeding practices.

Syrian refugees prefer to receive health services from institutions that provide services in their own language, therefore, they have no communication problems in RHCs in Turkey. On the contrary, structural barriers (e.g., irregular interpreter services, limited childcare options) impeding Syrian refugee women's access to healthcare were reported in Canada [53]. However, Syrian HCWs need further training on breastfeeding counseling and they are willing to take the training. During the study period, the breastfeeding counseling program was translated into Arabic in collaboration with the MoH and UNICEF, and the training is planned. At the same time, there is a need for culture-specific health services and female interpreters in maternity hospitals.

### Strengths and limitations

For the first time, the opinions and practices of HCWs, Syrian mothers, fathers, grandmothers about breastfeeding were collectively included in the qualitative survey. Having seven different respondent groups helped us to improve data validity with data triangulation and provide a rich understanding of breastfeeding challenges. The outcomes of this study will provide evidence for designing and implementing culturally acceptable and linguistically appropriate interventions for promoting breastfeeding for the Syrian refugees which will be for the benefit of the community at large in Turkey [54]. However, results may not be transferable to other countries.

## Conclusions

The results of this study are important in addressing the gap in breastfeeding where immediate interventions are needed [55]. The observed gaps in maternal knowledge should be taken into consideration for future interventions designed by HCWs, and health educators who should make a conscious effort to explain the benefits of breast milk to mothers, breastfeed on-demand, and the danger of prelacteal feeding and bottle-feeding [55]. Syrian refugees should be trained on age-appropriate feeding, and management of breastfeeding problems, scientific evidence on herbal teas, and family planning. Food support could be provided to lactating mothers. Continuity of girls' education and prevention of teenage marriages is needed.

## Data Availability

The data sets are available from WHO Turkey Country Office Refugee Health Program on request.
